# Dendritic cell subsets in cancer immunity and tumor antigen sensing

**DOI:** 10.1038/s41423-023-00990-6

**Published:** 2023-03-22

**Authors:** Annalisa Del Prete, Valentina Salvi, Alessandra Soriani, Mattia Laffranchi, Francesca Sozio, Daniela Bosisio, Silvano Sozzani

**Affiliations:** 1 Department of Molecular and Translational Medicine, University of Brescia, Brescia, Italy; 2 Humanitas Clinical and Research Center-IRCCS Rozzano, Milano, Italy; 3 Laboratory Affiliated to Istituto Pasteur Italia-Fondazione Cenci Bolognetti, Department of Molecular Medicine, Sapienza University of Rome, Rome, Italy; 4 IRCCS Neuromed, Pozzilli, IS Italy

**Keywords:** Dendritic cell subsets, Tumor microenvironment, Migration, Innate immune sensing, Immune cell death, Tumor-derived extracellular vesicles, Dendritic cells, Tumour immunology

## Abstract

Dendritic cells (DCs) exhibit a specialized antigen-presenting function and play crucial roles in both innate and adaptive immune responses. Due to their ability to cross-present tumor cell-associated antigens to naïve T cells, DCs are instrumental in the generation of specific T-cell-mediated antitumor effector responses in the control of tumor growth and tumor cell dissemination. Within an immunosuppressive tumor microenvironment, DC antitumor functions can, however, be severely impaired. In this review, we focus on the mechanisms of DC capture and activation by tumor cell antigens and the role of the tumor microenvironment in shaping DC functions, taking advantage of recent studies showing the phenotype acquisition, transcriptional state and functional programs revealed by scRNA-seq analysis. The therapeutic potential of DC-mediated tumor antigen sensing in priming antitumor immunity is also discussed.

## Introduction

Dendritic cells (DCs) are antigen-presenting cells (APCs) that play a crucial role in bridging innate and adaptive immune responses [1]. DCs patrol the local environment through the extensive expression of membrane and cytosolic receptors that recognize different types of danger signals, including pathogens and altered cells, such as tumor cells [2]. Upon antigen uptake, activated DCs, as professional APCs, process and present self and nonself antigens to naïve T lymphocytes, priming antigen-specific immune responses and regulating both tolerance and immunity [3].

DCs are considered central components of the tumor microenvironment (TME) and can promote antitumor T-cell responses [4]. However, an immunosuppressive TME can affect DC effector functions, altering DC phenotype and promoting dysfunction and tolerogenicity. These outcomes are mediated through different mechanisms involving soluble mediators as well as cell-to-cell contact [5, 6]. Notwithstanding the positive response to immune checkpoint inhibitors (ICIs) in the clinic, a complete clinical response is observed in only a small fraction of patients [7]. DCs have been shown to play critical roles in the therapeutic response to ICIs and represent attractive targets for cancer immunotherapy [8–10].

DCs constitute a heterogeneous group of immune cells that can be classified into different subsets both in humans and mice according to their ontogeny, phenotypical features, tissue distribution and transcriptional profiles [2, 11, 12]. DCs are generally divided into conventional or classic DCs (cDCs), which include cDC1s and cDC2s and plasmacytoid DCs (pDCs). cDC1s specialize in intracellular antigen processing and presentation and in shaping antitumor immune responses by cross-presenting tumor-associated antigens to CD8^+^ T lymphocytes, which recognizes them via major histocompatibility complex class I (MHC I) signaling [13]. cDC2s efficiently present MHC II-associated antigens to CD4^+^ T cells, promoting Th1, Th2, and Th17 polarization [14]. A single-cell analysis revealed an additional level of complexity in DC heterogeneity via the identification of multiple cDC2 subsets, such as DC2 and DC3 [15, 16], whose developmental origin and functional properties need further investigation [17, 18]. Plasmacytoid DCs are the major producers of type I interferons (IFNs) and are involved mainly in antiviral and antitumor immune responses [19]. Finally, monocyte-derived DCs (moDCs) represent a DC subset of cells that differentiate in response to inflammatory stimuli and are recruited to inflammatory sites, such as the TME [20].

The functional role of DCs in cancer immunology has been discussed in-depth in recent reviews [4, 21, 22]. Here, we aim to summarize the dual and opposing roles of DC subsets as tumor-promoting and tumor-suppressing cells, with a particular focus on i) tumor environmental signals that dictate DC functional properties, including cellular stress and cell death signals; ii) the molecular mechanisms regulating DC subset migration into the TME; iii) novel clues elucidating tumor-associated DC biology that have been derived from single-cell transcriptional analysis; and iv) the therapeutic potential of DC sensing that primes adaptive immunity against tumors.

## Dendritic cell subsets in cancer

Based on their specialized functional properties, DC subsets can affect tumor development and progression via various mechanisms, depending on dynamic changes in the local milieu [4, 23, 24]. cDC1s, which are under the transcriptional control of IRF8, ID2 and BATF3, can be phenotypically distinguished on the basis of the preferential expression of the chemokine receptor XCR1 and the C-type lectin receptor DNGR-1/CLEC9A (CD370) [25, 26]. In humans, cDC1s are recognized by the expression of BDCA3 (CD141), while in mice, they cDC1s express the integrin CD103, which is in the migratory cell subset, and CD8α in a lymphoid-resident cell subset. cDC1s are critical for the generation of antitumor immune responses due to their ability to cross-present tumor antigens derived from necrotic and apoptotic tumor cells [27]. cDC1 depletion in *Batf3***-**deficient mice impaired the capacity of the mice to reject transplantable immunogenic tumors and led to compromised T-cell-mediated responses to tumor immunotherapy, including ICI treatments [8, 10, 28, 29]. Furthermore, several studies have reported a positive correlation between cDC1 tissue density, a therapeutic response and patient overall survival in different solid tumors [30].

cDC2 differentiation is driven by the transcription factors IRF4, ID2, ZEB2 and NOTCH2/KLF4 [12], and this subset is identified by the expression of BDCA1 (CD1c), SIRP-α (CD172a, and CLEC10A (CD301) in humans and CD11b in mice [31, 32]. The role of cDC2s in cancer immunology is less established than that of other DCs, probably due to their heterogeneity and to the lack of markers enabling their clear identification. These cells are considered to be key inducers of CD4^+^ T helper cell responses for efficient presentation of MHC II-associated tumor antigens [22]. Following antigen uptake, cDC2s migrate to tumor-draining LNs, where they directly prime CD4^+^ T cells or transfer antigens to resident DCs [33]. Infiltration of CD4^+^ T cells in the TME has been correlated with the ratio of cDC2s to T regulatory cells (Tregs). A higher frequency of cDC2s correlated with greater CD4^+^ T-cell tumor infiltration [34]. The antitumor function of CD4^+^ T cells depends not only on their ability to directly activate cytotoxic T lymphocytes (CTLs) but also on the activation of macrophages and NK cells that they induce through the secretion of INFγ [22, 23]. In terms of prognostic value, the functional relevance of cDC2s within the TME is still unclear [35–37]. cDC2s constitutes heterogeneous cell subsets that include the recently identified DC3s (discussed in a later section).

pDC differentiation is guided by the transcription factors IRF8, RUNX1, and TCF4 [38]. pDCs are recognized in humans by their expression of BDCA2/CLEC4C (CD303), CD123 and BDCA4 (CD304), while in mice, they are identified by the surface markers Siglec-H and B220 [39]. As the main producers of type I IFNs, pDCs induce cDC1 maturation within the TME and to potentiate CD8^+^ T-cell and NK cell effector functions [40]. A pDC subset expressing high levels of OX40L has been identified in human head and neck carcinoma and shown to trigger potent tumor antigen-specific CD8^+^ T-cell responses together with those induced by conventional DCs [41]. The antitumor function of pDCs is also mediated by the expression of the cytotoxic molecules granzyme B and TRAIL [42]. On the other hand, pDCs foster tumor growth through the expression of immunosuppressive molecules, such as PD-L1, ICOSL, and indoleamine 2,3-dioxygenase, or the promotion of Treg expansion [42]. In addition, depending on the dose and timing, type I IFNs can promote cancer progression and immune evasion [43]. Indeed, in several tumors, dysregulated and tolerogenic pDCs have been associated with poor prognosis [44, 45]. MoDCs are associated with an inflammatory environment and share phenotypic and functional features with monocytes and cDC2s. MoDCs are described as dependent on IRF4 that can cross-prime CD8^+^ T cells [46]. The presence of moDCs has been recently associated with therapeutic responses to anti-PD-1 checkpoint blockade-only and combination therapies [47].

The balance between the intrinsic properties of DC subsets and their plasticity in adapting to the local environment characterizes their functional polarization in either promoting or inhibiting tumor development.

## New insights into tumor-associated DC biology

**Fig. 1:**
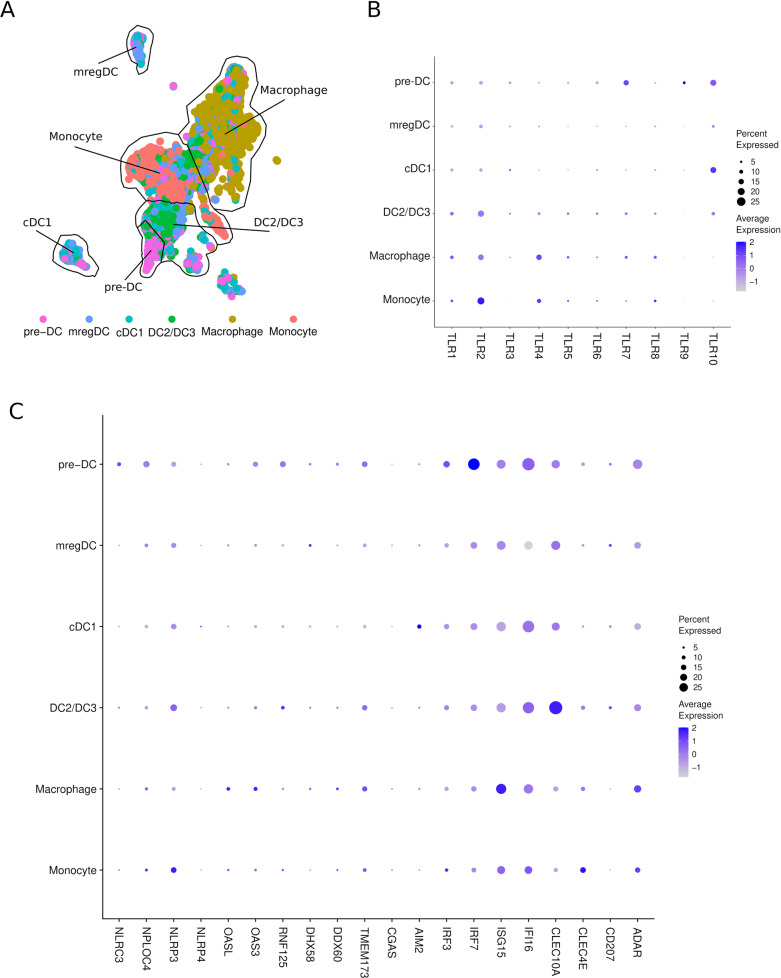
Pathogen-sensing machinery expression in human DC populations in the human myeloid scRNA atlas. **A** Visualization of PhenoGraph clusters on an MNP-VERSE UMAP plot following the coordinates presented by Mulder et al., 2021. Briefly, MNP-VERSE was downloaded from https://github.com/gustaveroussy/FG-Lab, and the data were explored with Seurat v4.2 (Hao and Hao et al. 2021, 10.1016/j.cell.2021.04.048). For simplicity, monocyte and macrophage clusters were unified. Bottom left, cells derived from cells enriched in cell cycle-related genes were unlabelled. **B** DotPlot presenting the normalized expression of TLRs from the MNP-VERSE. **C** DotPlot presenting the normalized expression of dsDNA machinery sensing from the MNP-VERSE

The constant improvement of scRNA-seq techniques and accompanying bioinformatics tools has led to a market increase in the number of studies in which cancer-specific immune cell ecosystems are explored [48]. Multiple groups have mapped tumor-infiltrating DCs in an unbiased manner, as they did not require a priori definition of protein markers. The profiles of DCs infiltrating different tumor isotypes is now available; these DC-infiltrated tumors include melanoma [16, 49], hepatocellular carcinoma [50], head and neck squamous cell carcinoma [51], non-small cell lung cancer [36, 52, 53], cutaneous squamous cell carcinoma [54], ovarian cancer [52], breast cancer [52] and colorectal cancer [52]. Although each study adopted a specific tissue dissociation protocol, scRNA-seq technology, and bioinformatics pipelines and considering the extreme heterogeneity of these tumor samples, the results showed conserved profiles among tumor-infiltrating DCs [55, 56] and conserved DC populations between human and murine systems [36, 53, 57] (Fig. 1). Since mouse animal models are commonly used in cancer immunology, their reliable representation of tumor-infiltrating DCs represents a relevant factor for comparison analysis in clinical studies. In fact, although the profiles of tumor-infiltrating DCs have been characterized in multiple cancers, most of the related studies were performed with untreated subjects [55], leaving the characterization of DC heterogeneity in patients undergoing anticancer therapies relatively unexplored.

scRNA-seq studies have led to the identification of a multitude of novel DC clusters on the basis of their transcriptional state [15, 36, 50, 53, 58]. However, the extreme single-cell resolution achieved blurred the distinction between the identification of a novel cell subset and the characterization of cells in a certain state. Therefore, each novel population needs to be extensively validated at the protein level [48]. One example of a recently characterized immune cell subset is the DC3 population. Previous reports demonstrated the breadth of human peripheral blood cDC2s, where two cDC2 subsets were identified in inflammatory diseases, namely, CD5^+^CD163^–^CD14^–^ cDC2s and CD5^–^CD163^+^CD14^+^ cells, which have been renamed cDC3 [15, 59]. Results from scRNA-seq studies have indicated that cDC2s constitute a heterogeneous group of cells consisting of bona fide cDC2s and DC3s [15, 56, 58]. DC3s are the circulating precursors of inflammatory DCs, as they are associated with an inflammatory gene signature [15, 56, 59]. DC3s develop along a different lineage promoted by GM-CSF, independent of CDPs, in a mandatory step for the development of both cDC1s and cDC2s [17, 18]. Since they exhibit distinct transcriptional and protein profiles and activate a different development pathway compared to those in other cDC subsets, cDC3s are classified as a novel circulating DC subset [56]. To date, there is no strong evidence that the DC3 lineage is carried in other species, and further studies are needed to fully characterize the roles of DC3s in pathophysiological conditions. Inflammatory DCs (bona fide DC3s) were identified in tumor ascites from patients with breast and ovarian cancer [20]. These cells can induce CD4^+^ T-cell responses and IL-17 production, but they have not been in cancer-free lymph nodes, indicating the need for a cancer niche for their function [20, 22]. The heterogeneity of cDC2s is further sustained by the presence of a peculiar DCs subset resembling the DC3 population in viral infections. These cells express CD64, a macrophage marker, and IRF8, a transcription factor also expressed by cDC1s, and optimally prime both CD4^+^ and CD8^+^ T-cell-mediated immunity [60, 61]. The role of this inflammatory type of cDC2 in tumors still needs to be defined.

Within the TME, cDCs have been shown to acquire a common gene program characterized by the expression of immunoregulatory genes (e.g., *CD274/*PD-L1*, PDCD1LG2/*PD-L2 and *CD200*), *LAMP3* and *CCR7*, thus informing the naming of these tumor-specific cells mregDCs or LAMP3^+^CCR7^+^ cDCs [22, 53]. In contrast to DC3s, mregDCs do not represent a characterized cell subset but in contrast represent an activated cell type. Different scRNA-seq cancer studies mapped the same gene signature in both infiltrating cDC1s and cDC2s in mice and humans [36, 50, 53, 62, 63]. Despite sharing the same cell state, only cDC1-like mregDCs have been associated with the expression of IL12B [63], thus suggesting that its DC lineage features are preserved even after the acquisition of the “mreg” gene program. Notably, recent evidence has revealed that mregDCs are evident not only in cancer but also in other pathological tissues [55], including human psoriatic skin, where they prime T-cell-driven inflammation [55]. These findings suggest that the “mreg” gene program may be interpreted as a tissue-induced maturation signature not a cancer-related transcriptional state [22]. Additional studies are needed to fully characterize the expression program of DCs in health and diseases.

## Innate sensing by DCs in tumors

Innate immune sensing is critical for the recognition of cancer cells by the immune system in the early and late stages of disease development and conventional cancer therapies. Similar to their activation in the immune response to infections, DCs must be appropriately activated to trigger T-cell responses against cancer. Different innate immune sensing pathways are relevant to cancer biology. Mammals are equipped with several distinct classes of pattern recognition receptors (PRRs), which recognize pathogen-associated (PAMPs) or damage-associated (DAMPs) molecular patterns. In the TME, immature DCs are activated via PRRs, which, upon recognition of DAMPs released by injured or stressed cells under noninfectious conditions, trigger metabolic changes in DCs [64]. Indeed, augmented cellular turnover may result in increased cell stress and release of tumor-derived DAMPs [65]. In addition, conventional therapies may reinvigorate the production of damage signals, activating host innate immunity through PRR sensing functions [66]. One mechanism for detecting cancer and developing inflammation in a sterile environment involves the release of molecules that serve as alarmins, which alert host cells of a dangerous condition by triggering the innate immune system to eliminate most incipient cancer cells. Despite the important role of DAMPs in initiating the antitumor response, recent studies have shown that the abnormal persistence of these DAMPs in the tumor microenvironment is the basis of tumor progression.

**Fig. 2:**
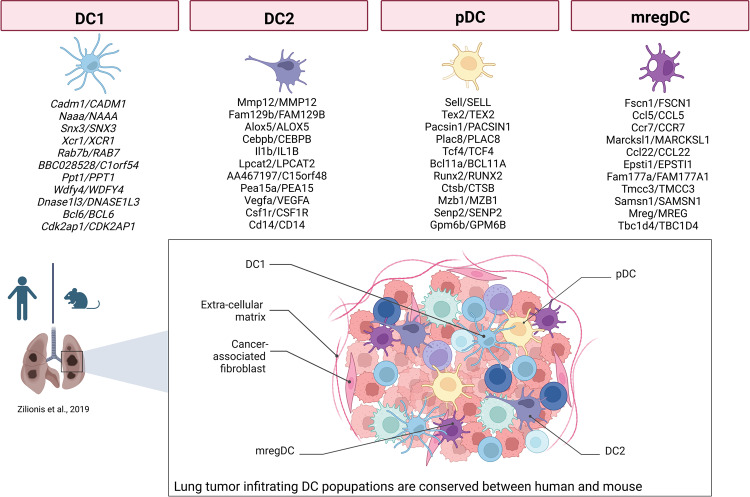
Lineage markers conserved between human and murine lung tumor-infiltrating DCs. Top panel, list of the 10 most highly expressed marker genes shared between human and mouse DC populations based on comparisons described in Zilionis et al., 2019. DC3s are missing due to the lack of a known homolog in mice. Bottom panel, schematic representation of the lung tumor microenvironment showing tumor-infiltrating DC populations. Adapted from “Tumor Microenvironment”, by BioRender.com (2022). Retrieved from https://app.biorender.com/biorender-templates

Each distinct DC subset expresses a unique profile of PRRs, which elicit a different response by inducing highly nuanced immune responses [67] (Fig. 2). PRRs include Toll-like receptors (TLRs) and C-type lectin receptors (CLRs), which mainly recognize extracellular stimuli, and cytosolic PRRs, such as retinoic acid inducible gene-I (RIG-I)-like receptors (RLRs), which are critical mainly for RNA sensing molecules; DNA sensors (e.g., cGAS/STING, AIM2); and NOD-like receptors (NLRs), which are critically important for the sensing of intracellular pathogens or DAMPs such as high-mobility-group box-1 protein (HMGB1), adenosine triphosphate (ATP), nicotinamide adenine dinucleotide (NAD+), and adenosine (ADO), which are released after tissue injury [68–72]. Recognition of DAMPs initiates the inflammatory response by activating the NF-κB, IRF3/7 or inflammasome signaling pathways, culminating in the production of proinflammatory cytokines, particularly type I IFNs. Signals originating from cancer cells are necessary for DC activation and effective priming of cancer-specific T cells, thereby launching the adaptive immune response to kill tumor cells [73]. Indeed, type I IFNs promote antigen retention and cross-presentation by DCs to enhance antigen-specific CD8^+^ T-cell responses [74, 75]. Notably, depending on whether DCs are exposed to PRR ligands and on the timing of tis exposure, T-cell functions are activated or suppressed [76]. Among PRRs, TLRs play active roles in cancer progression. TLR4 stimulation leads to phagosomal MHC class I enrichment through IkB-kinase (IKK)2-mediated phosphorylation of phagosome-associated SNAP23, which positively regulates cross-presentation of peptides derived from phagocytic cargo [77]. DCs enhance the efficiency of MHC-I loading via TLR engagement through the promotion of NADPH oxidase NOX2 activity [78]. TLR signaling may also drive antitumor effects by releasing cytokines into the TME to trigger antitumor immune responses or induce the apoptosis and programmed necrosis of tumor cells [79]. On the other hand, the overstimulation of TLR signaling may also drive cancer initiation/progression by provoking the release of proinflammatory cytokines (e.g., TNF-α, IL-1β and IL-6) or antiapoptotic, proliferative, and profibrogenic signaling molecules that contribute to the conversation of normal cells into tumor cells [80].

While DAMPs were initially considered to be exclusively released from necrotic cells, recent evidence suggests that specific forms of programmed cell death, such as necroptosis and immunogenic cell death (ICD) induced by anticancer therapies [81, 82], can also trigger DAMP secretion into the extracellular space. Over the years, anthracycline-induced cell death of tumor cells has been shown to cause the release soluble molecules, including HMGB1 and ATP [83], resulting in tumor elimination by tumor-specific T cells [84]. The interaction between HMGB1 and TLR4 controls antigen presentation by slowing the degradation of the phagocytic cargo and is one of the molecular mechanisms that dictates the chemotherapy-elicited antitumor immune response [85, 86]. TLR9, the first identified DNA-sensing receptor expressed in innate immune cells, is critical in mediating the accumulation, antigen uptake, lymph node migration, and maturation of tumor DCs after chemotherapy, as indicated by TLR9-deficient mice failing to present effective antitumor immune responses [87].

Several studies with humans indicated that nucleic acid (NA) sensors can be tumor suppressors and can be considered predictive biomarkers for certain types of tumors, presenting major implications for cancer immunotherapy [88]. Recent literature suggests that multiple tumor DNA-sensing pathways may be simultaneously involved in the stimulation of the antitumor immune response following chemotherapy in both animal models and cancer patients. One well-characterized signaling pathway is the STING pathway. Tumor-infiltrating DCs can sense tumor-derived DNA via cyclic-GMP-AMP synthase (cGAS) that, upon activation, catalyzes the synthesis of a noncanonical cyclic dinucleotide 2’3’- cGAMP, which binds to the adaptor STING, resulting in the recruitment and activation of the kinase TBK1 and transcription factor IRF-3; these events are essential to generate the robust type I IFN-mediated response needed for both spontaneous and treatment-induced antitumor immunity. In particular, STING activation results in the production of type I IFNs and maturation of Batf3-expressing DCs following the engagement of IRF3 and NF-κB [89]. Signaling through the STING pathway is the upstream link that triggers type I IFN production by DCs and facilitates effective cross-priming of tumor-specific CD8^+^ T cells in a variety of tumor models, including colon carcinoma, melanoma, and lymphoma models [90–92]. These findings provide a possible explanation for how antitumor T cells are primed in the absence of pathogen-derived innate stimuli. Importantly, various therapeutic approaches have been shown to require the DC-STING-IFN axis for full efficacy; these therapies include regimens of ionizing radiation and genotoxic agents [93]. In contrast to MyD88-, TRIF- and TLR-knockout mice, in which no defects were observed in spontaneous cross-priming of tumor-specific T cells, STING- and IRF3-deficient mice showed the defective accumulation of antitumor-specific CD8^+^ T cells in the seminal experiments performed by Woo et al. [90]. STING was also required in a S*TING-*deficient mouse model of colitis-associated colorectal cancer, which showed high susceptibility to tumor formation [94]. In a mouse model of intracranial glioma with type I IFN receptor deficiency, the acceleration of gliomagenesis was associated with an increased infiltration of myeloid-derived suppressor cells (MDSCs) and Tregs along with decreased infiltration of DCs and CTLs [95]. In the same model, a loss-of-function mutation in STING resulted in the reduced production of type I IFN in myeloid cells, impairing tumor growth control by the immune system [96]. With a series of murine models, it has been demonstrated that shortly after tumor challenge in vivo, type I IFN production was required for spontaneous cross-priming of tumor antigen–specific CD8^+^ T cells [97]. The CD8α^+^ DC subset, which has been shown to play a critical role in the development of tumor-specific immune responses [98], specifically requires type I IFNs to prime protective tumor-specific immune responses in vivo [99].

Despite all the findings discussed thus far, there is also evidence that chronic activation of the STING pathway can promote tumorigenesis due to the persistent production of inflammatory cytokines and recruitment of phagocytes, which create an inflammatory milieu that promotes tumor development [100, 101]. Moreover, STING can foster tumor progression and metastasis by modifying the TME, making it tolerogenic through increased IDO production [102]. In a model of lung cancer, lactate in the TME caused loss of DC function and failed to prime antitumor responses because the action of type I IFNs downstream of TLR3 and STING was inhibited [103]. Thus, an appropriate balance in STING pathway activation and inhibition may be required for optimal antitumor effects. STING agonists, such as 5,6-dimethylxanthenone-4-acetic acid (DMXAA), support DC maturation and cross-presentation of antigens, exhibiting significant antitumor efficacy in several mouse models [104], but this agonist was unable to bind human STING [105].

Increasing evidence has shown that DCs recognize NAs released from stressed or dying cancer cells via RLRs to initiate innate immune responses in the tumor microenvironment. Initially, type I IFN production promotes the activation and maturation of DCs, which further cross-prime tumor-specific T cells to control tumor growth [90]. Based on the role of NA sensing in antitumor immunity, cGAS-STING and RIG-I/MDA5 agonists have been developed for cancer immunotherapy, even though some controversial studies have also shown that inappropriate activation of STING and RIG-I signaling can contribute to a suppressive TME and promote tumor growth and metastasis [102].

Absent in melanoma 2 (AIM2) is a cytosolic dsDNA sensor that has been extensively studied because of its role in inflammasome assembly and subsequent activation of caspase 1, which in turn, leads to the generation of the mature forms of IL-1β and IL-18 [106]. AIM2 exhibits an inhibitory effect on the STING pathway, suggesting that induction of pyroptosis by the AIM2 inflammasome normally dampens STING pathway activation in response to cytosolic DNA [107]. Moreover, AIM2 expression in human melanoma-infiltrating DCs correlated with tumor progression. In particular, it was demonstrated that the effect of a DC vaccination was enhanced by AIM2-deficient DCs and that this efficacy depended on STING–type I IFN signaling [108]. Activation of the NLRP3 inflammasome within DCs is decisive for the immune response against dying tumor cells, providing a previously unknown link between the innate and acquired immune systems [83]. Recently, C-type lectin receptors (CLRs) were included in the PRR family because they regulate the antitumor immune response. Indeed, carcinoembryonic antigen (CEA), a widely expressed tumor-associated antigen (TAA), is specifically recognized by DC-SIGN, a C-type lectin receptor on the surface of dendritic cells [109]. Signaling mediated by this receptor is required for endocytosis and antigen cross-presentation [110]. On the other hand, glycans, such as those with a mannose structure, Lewis-type antigens, and mucin 1, which undergoes cancer-specific glycosylation changes, are components of tumor antigens that ligate CLRs, contributing to immunoregulatory effects [111].

## Tumor antigen presentation by DC subsets

Tumor antigen presentation by DCs is an essential process for the priming of antigen-specific cytotoxic CD8^+^ T lymphocytes [112]. Upon antigen uptake, DCs undergo a maturation process characterized by the upregulation of the costimulatory molecules CD80, CD86, and CD40; the production of proinflammatory cytokines; and CCR7-dependent migration to draining lymph nodes. These transcriptomic and epigenomic changes are strengthened by the formation of DC:T-cell immune synapses [113, 114]. cDC1s constitute the DC subset specialized in the cross-priming of tumor antigens at the early phases of lung tumor development, a process fine-tuned by the C-type lectin receptor CLEC9A [110] and the phosphatidylserine receptor TIM4, which is selectively expressed by lung cDC1s and mediates engulfment of cell-associated antigens, the induction of antitumor responses and efficacious immunogenic therapy [98, 115, 116]. In experimental tumor models, *Batf3*-competent cDC1s have been shown to be involved in the reactivation of circulating central memory T cells into antitumor resident central memory T cells, a process further promoted by anti-PD1 immunotherapy [117]. Tumor-resident cDC1s are crucial for regulating the trafficking of effector T cells to the TME through the secretion of CXCL9 and CXCL10, suggesting that cDC1-T-cell crosstalk is also evident at tumor sites [8]. On the other hand, migratory cDC2s are critical for the induction of antitumor CD4^+^ cell priming, both in vitro and in vivo [34]. However, this paradigm was recently challenged since cDC1s can also induce naïve CD4^+^ T-cell priming by presenting tumor antigens via the MHC II pathway. CD4^+^ T cells can in turn license, in a CD40-dependent manner, cDC1s to promote an efficient antitumor CD8^+^ T-cell response [118]. These findings suggest that cDC1s orchestrate antitumor responses by priming both CD4^+^ and CD8^+^ T cells.

Tumor antigens can also be delivered to resident lymph node DCs by migratory DCs. This was shown in an experimental model of melanoma in which antigens embedded in vesicles were released by both cDC1 and cDC2 and transferred to resident cells through close and sustained synapsis-like contacts between donor and recipient DCs [119].

In addition to their specialization in producing type I IFNs, tumor pDCs are generally considered tolerogenic cells, and intratumoral pDCs are frequently correlated with a poor prognosis [120]. However, activation of pDCs has also been associated with an effective antitumor immune response, suggesting the possibility that pDCs may present and activate CD8^+^ T cells through cross-presentation of tumor antigens [121]. Cross-presentation of antigens by pDCs may require the transfer of antigens to bystander cDCs through pDC-derived exosomes [122]. Although both cDC1s and cDC2s receive antigens from pDCs, only cDC1s are indispensable for pDC-mediated cross-priming [122].

Tumor-associated moDCs can serve as antigen-presenting cells as well, although they are less efficient in activating naïve antigen-specific T cells [123].

The presentation of tumor antigens by DCs is a crucial step for the induction of the effector T cell-mediated killing phase. The understanding of this process is becoming very important in the context of cancer immunotherapy and may lead to new opportunities for the development of targeted therapies. In the next sections, recently characterized mechanisms of antigen capture and cross-presentation by DCs in the TME and tumor-draining lymph nodes are further discussed.

## Role of ICD in DC activation

The Nomenclature Committee on Cell Death has recently defined immunogenic cell death as “a form of regulated cell death that is sufficient to activate an adaptive immune response in immunocompetent hosts” [124]. ICD represents a functionally unique response pattern that is initiated with the induction of organellar and cellular stress and ultimately results in cell death accompanied by the exposure, active secretion or passive release of different DAMPs [124, 125]. During ICD, DAMPs interact with PRRs that are expressed by immune cells, especially by DCs, to initiate a cell-activating cascade that culminates in the activation of both innate and adaptive immune responses [126].

**Fig. 3:**
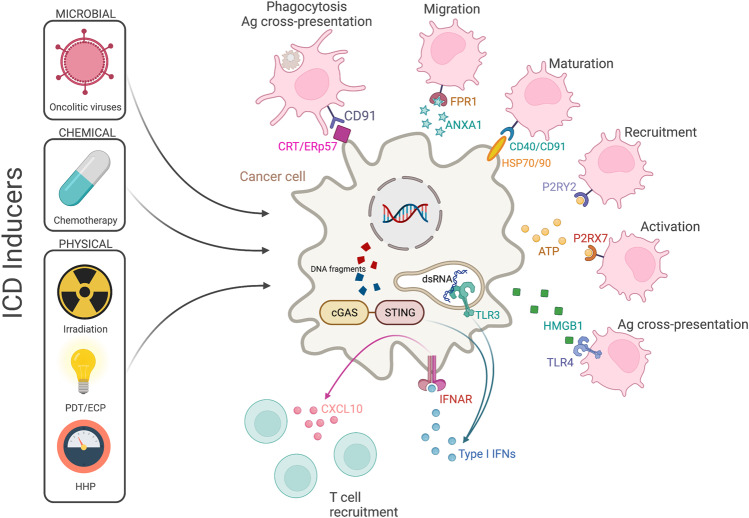
Role of immunogenic cell death in dendritic cell (DC) activation. In response to different inducers of ICD, tumor cells expose/secrete several damage-associated molecular patterns (DAMPs) that interact with DCs. PDT photodynamic therapy, ECP extracorporeal photochemotherapy, HHP high hydrostatic pressure, CRT calreticulin, ANXA1 annexin A1, FPR1 formyl peptide receptor 1, HSP heat-shock protein, P2RY2 purinergic receptor P2Y2, P2RX7 purinergic receptor P2X7, HMGB1 high-mobility group box 1, TLR Toll-like receptor, IFN interferon, IFNAR type I interferon receptor. Created with BioRender.com

ICD may provide a new strategy to increase the effectiveness of anticancer treatment since chronic exposure to TME-associated DAMPs may favor the activation of long-lasting antitumor immunity. The involvement of DCs in the immune response triggered by cancer cells undergoing ICD has been described in several studies [127, 128]. The findings suggest that the ability of different ICD inducers to stimulate an efficient antitumor T-cell response may depend DC activation in the TME. ICD inducers comprise conventional chemotherapeutics (i.e., anthracyclines, cyclophosphamide, oxaliplatin and other platinum derivates, but not cisplatin) [129]; selected targeted anticancer agents, such as the tyrosine kinase inhibitor crizotinib [130], the anti-epidermal growth factor receptor-specific monoclonal antibody cetuximab [131] and poly-ADP-ribose polymerase (PARP) inhibitors [132]; radiotherapies [133]; oncolytic viruses [134]; nanopulse stimulation [135]; and different physical induction strategies such as photodynamic therapy [136], extracorporeal photochemotherapy [137], high hydrostatic pressure [138] and various forms of ionizing radiation [139] (Fig. 3).

DAMPs released during ICD can localize to different cellular compartments [140]. DAMPs exposed on the cell surface include calreticulin (CRT) and heat-shock protein (HSP) 70 and HSP90; DAMPs secreted from dying cells into the extracellular space include the small metabolite ATP, the nonhistone chromatin-binding protein high-mobility group box 1 (HMGB1), and the cytoplasmic protein annexin A1 (ANXA1); DAMPs that appear during the end-stage degradation of ICD factors include mitochondrial components, DNA and RNA. CRT is a Ca^2+^-binding protein located mainly in the lumen of the ER and is exposed with its cofactor ERp57 on the plasma membrane in response to ICD inducers, providing a potent “eat me” signal to DCs [141]. The surface-exposed CRT/ERp57 complex binds low-density lipoprotein receptor-related protein 1 (LRP1, better known as CD91) on the DC membrane, promoting phagocytosis, tumor antigen cross-presentation and activation of antitumor CTLs. The CRT-CD91 interaction promotes the release of proinflammatory cytokines, leading to Th17 cell priming [142]. In patients with non-small cell lung cancer, CRT expression is correlated with a positive prognosis that is putatively associated with a higher infiltration of mature DCs and effector memory T-cell subsets [127]. Similar to CRT, HSP70 and HSP90 are exposed on the plasma membrane of dying cells, which facilitates their interaction with their respective receptors, CD40 and CD91, located on DCs [143, 144]. HSP70 induces DC maturation by promoting the upregulation of the costimulatory molecules CD86 and CD40, thereby increasing CTL responses. In addition, HSP70 interacts with TLR4 on DCs and induces the production of proinflammatory cytokines via NF-kB activation [145]. During ICD, ATP is released into the extracellular milieu where it is a “find me” signal for DC precursors and macrophages after binding to the metabotropic (P2Y2) purinergic receptor, facilitating the recruitment of APCs to the sites with active ICD [146]. ATP signaling mediated through the ionotropic (P2X7) purinergic receptor on the surface of DCs activates the NLRP3 inflammasome and leads to the subsequent secretion of IL-1β. These actions culminate with the activation of CD8^+^ T cells and IL-17-producing γδ T cells [83]. In addition, breast cancer patients carrying a loss-of-function allele of *P2RX7* showed unfavorable disease outcomes compared to individuals carrying the normal allele [83]. HMGB1 is released from cancer cells in the late stage of ICD, when both nuclear and plasma membranes lose their integrity [147]. Extracellular HMBG1 can bind different PRRs expressed by APCs, such as the receptor for advanced glycation end products (RAGE), TLR2 and TLR4 [148]. The binding of extracellular HMGB1 to TLR4 inhibits the fusion between phagosomes and lysosomes, which is involved in the cross-presentation of tumor antigens by DCs. In the absence of HMGB1 or TLR4, dying cells are regularly engulfed by DCs, resulting in cell degradation not antigen presentation [85]. Notably, *TLR4* loss-of-function polymorphisms have been associated with poorer clinical outcomes in response to chemotherapy in colon cancer and head and neck squamous cell carcinoma patients as well as in melanoma patients treated with an experimental DC-based vaccine [149, 150]. In ICD, after plasma membrane permeabilization, the liberation of ANXA1 guides DC to migrate to the proximity of dying cancer cells via a formyl peptide receptor 1 (FPR1)-dependent mechanism [151].

Finally, ICD is characterized by the de novo synthesis of type I IFNs triggered by the accumulation or delocalization of aberrant RNA molecules that are detected by endosomal TLR3 [152], as well as the formation of micronuclei and mitochondrial DNA, which promote the activation of the cGAS-STING pathway [153]. Type I IFNs exert potent immunostimulatory effects by enhancing the cytotoxic functions of both CD8^+^ T cells and NK cells [154] and promoting cross-priming by DCs [155]. Type I IFNs promote the production of CXCL10 (a chemoattractant for T cells) by cancer cells undergoing ICD via an autocrine signaling loop [156]. This cascade of events can drive robust tumor infiltration of myeloid and lymphoid cells. Notably, the attack of cancer cells by CTLs may also result in ICD, suggesting a mechanism of self-amplification that might facilitate antigen spread during the local response [157].

## Tumor-derived extracellular vesicles regulate DC functions in cancer

**Fig. 4:**
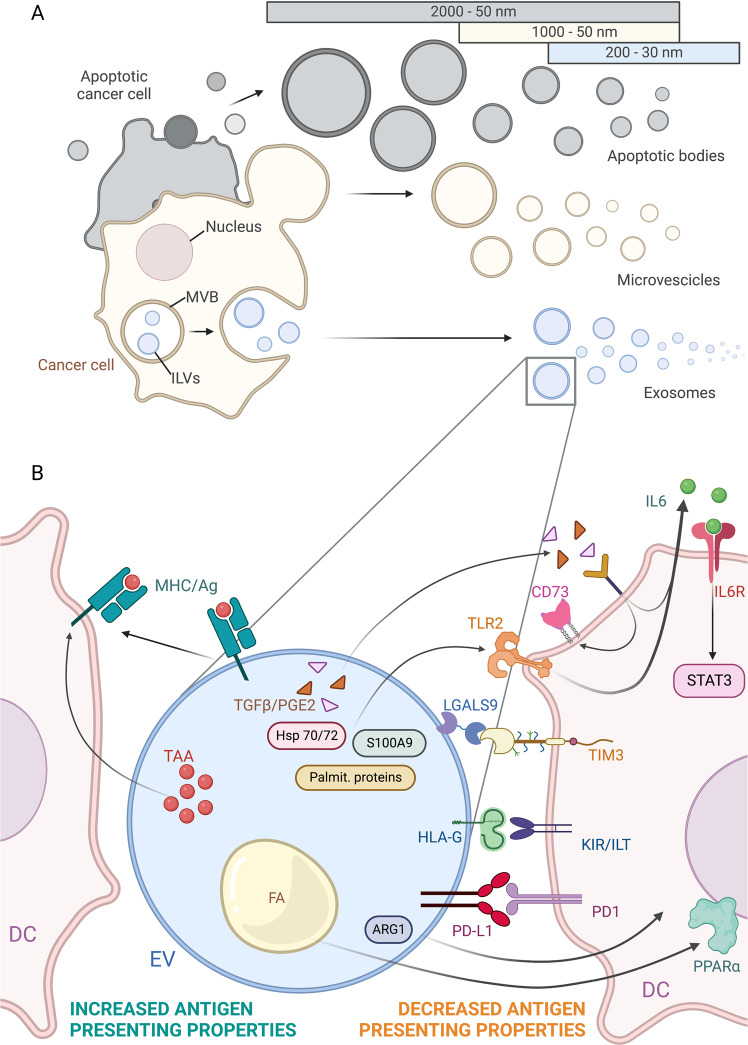
Tumor-derived extracellular vesicles (EVs) regulate the antigen-presenting properties of DCs. **A** EV subpopulations are represented based on size and origin. Exosomes are the most extensively studied tumor-derived EVs. Very few authors have addressed the role of different EV subpopulations in modulating DC biology in tumors. **B** Tumor-derived EVs have been shown to mediate two main and apparently contradictory functions in DCs, i.e., increased (depicted on the left) or decreased tumor antigen presentation (right side). The image summarizes the different regulatory mechanisms in a single EV different identified in specific tumor types and EV subpopulations. MVB multivesicular body, ILVs intraluminal vesicles, TAA tumor-associated antigen, FA fatty acid, palmit. proteins palmitoylated proteins, ARG1 arginase 1. Created with BioRender.com

Cells of virtually all organisms and tissues, including tumor and immune cells, release vesicles identified by double-leaflet lipid membranes and known as extracellular vesicles (EVs). EVs influence homeostatic processes as well as the development and progression of diseases, including cancer [158]. Hence, EVs represent promising therapeutic and diagnostic tools [159]. EVs include both exosomes and microvesicles, also known as ectosomes or microparticles, respectively [160]. Exosomes originate as intraluminal vesicles in the endosomal compartment that are formed upon inward budding of the endosomal membrane within the lumen of late endosomes (more often referred to as multivesicular bodies, MVBs) and are released by the fusion of MVBs with the plasma membrane [161]. In contrast, microvesicles are generated via direct budding (or shedding) from the plasma membrane of living cells [161]. A third EV subtype following this same biogenetic pathway is composed of apoptotic bodies that are exclusively released during apoptotic death (Fig. 4A). In general, exosomes and microvesicles differ in size: specifically, exosomes are often referred to as “small EVs” because their mean diameter is less than 150 nm, while microvesicles (and often apoptotic bodies) are called “large EVs”, because their diameter is at least 1000 nm. EVs can carry membrane, cytosolic and nuclear proteins, extracellular matrix proteins, metabolites, mRNA, several types of noncoding RNAs, including microRNAs, and DNA [159]. All these components have been identified in tumor EVs (TEVs), reflecting the composition of the tumor cells from which the vesicle was derived. Two main and apparently contradictory functions have been described for TEV and DC interactions: they either promoted or inhibited tumor antigen presentation (Fig. 4B).

Initially, TEVs received attention as potential stimulators of DC antigen-presenting activity because of their ability to transport tumor antigens such as Her2/Neu, Mart1, TRP, gp100, MHC-I molecules and DC-activating HSPs [162]. Indeed, DCs have been shown to take up TEVs, process tumor antigens and subsequently elicit antigen-specific CTL responses [162, 163]. There is also evidence suggesting that tumor cells may transfer MHC/antigen complexes to DCs, a process known as cross-dressing [164]. From this perspective, TEVs appear to be privileged carriers that deliver antigens and immunostimulatory molecules to DCs in the context of DC-based vaccination [165], with TEV-pulsed DCs showing better antitumor efficacy than DCs pulsed with tumor lysates [166].

However, several lines of evidence show that TEVs may also reduce the antitumor function of DCs by inducing the differentiation of MDSCs. TEVs from melanoma cell lines or plasma from advanced melanoma patients inhibited the differentiation of human moDCs in vitro, inducing the acquisition of a phenotype corresponding to that of MDSCs [167]. Similarly, murine DCs treated with TEVs from mammary adenocarcinoma [168] have been associated with increased tumor growth. This effect depended on STAT-3 signaling, a pathway previously identified as exerting inhibitory effects on DC differentiation from CD34^+^ bone marrow progenitors. STAT-3 is activated by IL-6 directly released from MDSCs and stimulated by TEVs containing prostaglandin E2 and TGF-β. TEVs may also induce MDSCs because of their Hsp70 and Hsp72 content, triggering STAT-3 activity in a TLR2/MyD88-dependent manner through autocrine production of IL-6 [169]. In addition, the treatment of MDSCs obtained from cancer patients with a drug inhibiting exosome formation exhibited a reduced suppressor functions. Finally, TEVs derived from CD105^+^ cancer stem cells inhibited human DC differentiation by carrying HLA-G, a nonclassical MCH-I molecule known to engage inhibitory receptors expressed on T cells, NK cells and DCs [170, 171].

In addition, TEVs carrying PD-L1 also directly inhibit antigen presentation by DCs through the engagement of the inhibitory receptor PD-1, as shown in lung carcinoma and breast cancer cell lines [172]. Moreover, TEVs isolated from the cerebrospinal fluid of glioblastoma multiforme patients were separated based on their size [173]. Only medium and small EVs were efficiently taken up by DCs, and only a small fraction inhibited antigen presentation, and this effect was independent of the content of tumor-associated antigens. These vesicles were enriched with LGALS9, the ligand of the DC-expressed immune checkpoint TIM3. Experiments with LGALS9-KO mice revealed a sustained inhibitory role in tumor antigen presentation and the generation of long-lasting immunity played by this protein. Additionally, TEVs from melanoma cells were shown to alter DC functions in vitro [174], a finding confirmed recently in a study on TEVs recovered from human afferent lymphatic fluid drained from patients with skin melanoma [175]. In a mouse model of ovarian carcinoma, TEVs were shown to suppress the proliferation of CD4^+^ and CD8^+^ T cells in vitro and in vivo [176]. In this tumor type, TEVs carried arginase-1 (ARG1), an immunosuppressive molecule present in the tumor microenvironment that leads not only to the depletion of ʟ-arginine, a nutrient required for T-cell expansion but also to proper antigen presentation by DCs [177]. Notably, recombinant ARG1 did not inhibit T-cell proliferation, probably due to the fast degradation of the free enzyme in vivo, while EV-associated ARG1 might be protected from degradation by the EV membrane [176]. Another proposed mechanism underlying the inhibition of the DC-mediated CTL antitumor response depends on the inhibition of IL-12 and TNF-α production by ATP. This effect was realized via the upregulation of CD73 (an ecto-5′-nucleotidase that converts AMP to adenosine) in DCs in response to TEV-associated PGE2 exposure in prostate cancer [178]. Finally, TEVs have been shown to induce DC immune dysfunction by transferring fatty acids, which induce the expression of the peroxisome proliferator-activated receptor (PPAR) in DCs. The increase in both the biogenesis and oxidation of fatty acids induces mitochondrial oxidative phosphorylation with subsequent DC function impairment [179]; this is the first evidence showing DC metabolic reprogramming by TEVs.

## Molecular pathways regulating DC subset migration in tumors

The recruitment of DC subsets to the tumor microenvironment is regulated by the local production of chemotactic factors produced by both tumor and stromal cells, including immune cells [10, 23]. DC subsets, in particular the cDC1 subset, migrate to tumor-draining lymph nodes and present tumor antigens to naïve T cells via a CCR7-dependent mechanism [113, 180]. In lymph nodes, the bioavailability of the CCR7 ligands CCL19 and CCL21 is controlled by the atypical chemokine receptor ACKR4, which has been shown to regulate DC trafficking [181, 182].

DC precursors are attracted to the TME via the local production of growth factors, such as FLT3-L, and chemokines (e.g., CCL3), which mediate the in situ expansion of cDCs [10, 183]. The presence of cDC1s in the TME has been correlated with the presence of NK cells and is considered a positive prognostic factor in melanoma, glioblastoma and neuroblastoma patients [10, 184]. Moreover, CCL5 and XCL1 produced by tumor-infiltrating NK cells and innate lymphoid cells promoted the recruitment and activation of XCR1^+^ cDC1s, fostering NK-cDC1 axis-related control of tumor growth [25, 185, 186]. Immunoreactive tumors are characterized by the expression of CXCL9, CXCL10 and CXCL11. These DC-derived chemokines can recruit both NK and activated T cells to promote the antitumor response [184]. Indeed, the disruption of the NK-cDC1 chemotactic axis by tumor-derived PGE2 inhibited NK cell, cDC1 and effector T-cell infiltration in melanoma, as part of the programmed tumor evasion from the innate immune response [187]. The TME may also activate DCs to secrete chemokines that act on subsets of NK cells that are inefficient in antitumor immunity as an additional mechanism contributing to immune evasion. For instance, in lung and breast cancer, it has been reported that the production of CXCL9 and CXCL10 preferentially recruits CD56^bright^ cells, not the more cytotoxic CD56^dim^ NK cells [188].

The tumor-derived chemokine CCL4 has been shown to contribute to the recruitment of CCR5^+^ cDCs to the TME [189]. In addition, a role for CXCR3 in the trafficking of precDC1s to promote the local production of CXCL9 in melanoma tumors has been reported [190]. In mice, overexpression of CCL20 and CXCL14 was also shown to play an important role in attracting myeloid DCs to induce effective tumor suppression [191]. In melanoma, tumor cells recruit CXCR4-expressing pDCs in response to CXCL12 [192]. Another mechanism for pDC recruitment into tumors involves the IL-3-mediated upregulation of the chemokine receptor CCR6, which recruits pDCs through the CCR6/CCL20 axis [193].

Recently, the interest in the role of damage-associated molecular patterns (DAMPs) released by dying tumor cells as licensing signals that lead to DC activation and migration has increased [194]. ICD may contribute to the release of DAMPs or exposure of DAMPs on the surface of dying cells. One of the DAMPs released by dying cancer cells is ANXA1. ANXA1 is a ligand of formyl peptide receptor 1 (FPR1), a pattern recognition receptor that is expressed mostly in myeloid cells, including DCs. Cancer patients bearing mutations in FPR1 showed a selective disadvantage with regard to their prognosis. This has been documented for breast cancer in the context of adjuvant anthracycline-based chemotherapy, as well as in colorectal cancer patients in the context of adjuvant oxaliplatin-based chemotherapy, possibly due to defective TME infiltration by DCs [151]. Among many molecules, ATP released by tumor cells induced by chemotherapy has been reported to recruit inflammatory DCs and enhance antitumor immunity [195]. In a model of in situ ICD, tumor-infiltrating CD103^+^ cDC1s and CD103^-^ cDC2s showed accelerated turnover within the tumor and emigrated to draining lymph nodes mediated via the ATP/P2X7R and HMGB1/TLR4 signaling pathways, leading to an increased CD8^+^ T-cell antitumor response and the suppression of secondary tumor growth [196]. Finally, the release of calreticulin has been associated with DC infiltration into tumors, DC maturation through the secretion of TNF-α and CCL19 mediated *via* TLR4-MyD88 signaling, and a positive prognosis in NSCLC patients [197].

MoDC recruitment into the TME has been reported to depend on the CCR6/CCL20 axis. In a murine lung cancer model, a Th17 cell transcription factor RORγT agonist showed antitumor efficacy and increased the anti-PD-1 therapeutic response by promoting the secretion of CCL20 and the recruitment of moDCs [198].

## Immunosuppressive role of the TME on DC functions

The immunosuppressive TME causes several alterations in DC functions: 1) elimination of functional DCs; 2) suppression of key DC functions; 3) generation of tolerogenic and immunosuppressive cells; and 4) prevention of direct contact between DCs and tumor cells by downregulating the production of chemokines that act on DCs. All these mechanisms compromise antitumor immunity, supporting tumor progression.

Within the TME, several tumor- or stromal-derived mediators induce DC dysfunction and alter DC differentiation, maturation, antigen presentation and longevity [199]. One of the first cytokines shown to have an inhibitory effect on DC functions was IL-10. IL-10 converts immature DCs into tolerogenic DCs by decreasing the expression of costimulatory molecules [200]. In a breast cancer model, IL-10 was shown to inhibit the production of IL-12 by tumor-infiltrating CD103^+^ cDC1s, inhibiting T-cell responses [201]. IL-6 levels in the TME and in the sera of cancer patients have been correlated with poor disease outcomes [202]. IL-6 is a potent inhibitor of DC maturation because it downregulates both MHC-II molecule and CCR7 expression through STAT3 activation [203]. In the TME, DCs are induced to produce both IL-6 and IL-10, contributing to the generation of an immunosuppressive microenvironment [204]. VEGF is another tumor-derived factor shown to inhibit DC functions, including DC antigen presentation [205]. VEGF is produced by several types of tumors, and increased levels of this cytokine have been associated with a poor prognosis in cancer [206]. Several studies have revealed that VEGF significantly affects DC differentiation from precursors [207], and elevated levels of VEGF have been reported to correlate with a reduction in the numbers of infiltrating and circulating DCs [208].

Among the immunosuppressive factors produced by tumor cells, PGE2 and TGF-β1 promote the upregulation of PD-L1 by DCs, turning them into immunosuppressive cells [209]. CD103^+^ DCs from tumor-draining lymph nodes express increased levels of PD-L1, and blockade of PD-L1 and PD-1 mitigates DC dysfunction in terms of TNF-α, IL-12, and IL-1β production and enhances T-cell-stimulatory capacity [10]. TGF-β has been described as the main factor critical for the acquisition of a pDC immunosuppressive phenotype because it is crucial for the inhibition of TLR9-induced IFN-α production [210]. In vitro, PGE2 and IL-6 released by melanoma cells converted cDC2s into CD14^+^ cDCs, which were characterized with an immunosuppressive phenotype [211]. Notably, tumor-infiltrated CD14^+^ cDCs express high levels of markers that are characteristic of tumor-associated macrophages (TAMs), such as CD206, MerTK and CD163, suggesting that these cells exhibit TAM-like protumoral functions [211]. PGE2 has also been shown to induce the suppression of antigen presentation by DCs [212] and to modulate NK cell-mediated recruitment of cDC1s to the TME by reducing NK cell viability and chemokine production [185]. The PGE2-EP2/EP4 axis promoted the expression of NF-κB-regulated proinflammatory genes in myeloid cells, eliciting immunosuppression by driving the mregDC (mature DCs enriched in immunostimulatory molecules) [53]-Treg axis, thereby increasing Treg recruitment and activation in tumors [213]. Targeting cyclooxygenase (COX) in melanoma cells inhibited PGE2 production and promoted the accumulation of cDC1s and NK cells within tumors [61]. This COX inflammatory signature was found to be conserved in human melanoma biopsy samples, where PGE2 mRNA levels negatively correlated with CD8^+^ T infiltration [61].

Although lipid bodies have been shown to be required for the regulation of exogenous antigen cross-presentation to CD8^+^ lymphocytes [214], recent evidence suggests the abnormal accumulation of lipids in DCs is an additional mechanism that contributes to DC dysfunction in cancer [215]. Consistent with these findings, the constitutive activation of the ER stress response factor XBP1 in tumor-associated DCs has been shown to be critical for the induction of a triglyceride biosynthetic program leading to lipid accumulation and reduced antitumor immunity [216]. Oxidized lipids inhibit cross-presentation by sequestering the chaperone protein HSP70 and reducing the translocation of the MHC-I-peptide complex to the plasma membrane [217].

Tumor-derived oxysterols have been shown to downregulate CCR7 expression, thereby inhibiting DC migration to secondary lymphoid tissues and suppressing the generation of an antitumor immune response [218].

Tumor cells produce high levels of lactic acid that suppress IL-12 production and inhibit DC-dependent antigen presentation in vitro and in vivo via GPR81 signaling [103, 219]. In a breast cancer model, lactic acid inhibited type I IFN expression and promoted indoleamine 2,3 dioxygenase (IDO)-dependent Treg generation by pDCs [220]. Notably, the upregulation of IDO in tumor-associated DCs has been correlated with a regulatory DC phenotype. IDO-positive DCs inhibit the activity of CD8^+^ T, NK and plasma cells and contribute to the differentiation of Treg cells through conversion of 1-tryptophan, an amino acid essential for T-cell responses, into 1-kynurenine [221]. In a transgenic melanoma experimental model, a melanoma-derived Wnt5a ligand was shown to upregulate the expression and activity of IDO by local DCs in a β−catenin-dependent manner, thus promoting the differentiation of Tregs [222]. β-catenin-expressing tumors led to a reduction in CCL4 secretion, which prevented CD103^+^ cDC1 recruitment to the TME [189].

In addition to an acidic microenvironment, tumor progression generates regions of hypoxia that promote the expression of hypoxia inducible factor-1α (HIF-1α). HIF-1α is a negative regulator of pDC differentiation in vitro and in vivo [223]. In head and neck squamous cell carcinoma (HNSCC), a hypoxic TME promoted pDC migration through the HIF-1α/CXCL12/CXCR4 pathway and induced the acquisition of a tolerogenic pDC phenotype, which was characterized by defective production of IFN-α, reduced antigen presentation abilities, and expansion of Treg cells [224]. In cervical cancer, hypoxia upregulated HMGB1 release, leading to TLR9-mediated inhibition of pDC maturation and cytokine secretion and the generation of tolerogenic pDCs [225]. HMGB1 released by dying tumor cells also inhibited antitumor immune responses by competing with nucleic acid binding to TIM3, a receptor expressed by intratumoral DCs [226]. Tumor-associated hypoxia also promotes the accumulation of extracellular adenosine. Adenosine promotes a tolerogenic phenotype, mediated through multiple receptors [227] and the recruitment of immature pDCs and inhibits cytokine secretion [228].

In addition to promoting immune tolerance, DCs favor tumor growth and progression through the production of proangiogenic mediators that promote neovascularization, an essential aspect of tumor growth and metastasis. In vitro, human cDCs produce biologically active VEGF-A [229]. Consistent with this finding, cDC2s and interdigitating DCs, but not pDCs, are the major sources of VEGF-A in inflamed secondary lymphoid organs [230]. From a molecular point of view, VEGF-A production by DCs requires the activation of three transcription factors, namely, CREB, HIF-1α and STAT3 [230, 231]. Inflammatory agonists activate HIF-1α and STAT3, while CREB phosphorylation is induced by the autocrine/paracrine production of PGE2 [230, 232]. In addition to VEGF-A, DCs secrete other proangiogenic factors, such as FGF2 and ET-1 [233]. DCs are also important sources of chemokines that can modulate angiogenesis via direct or indirect mechanisms. DCs express the pro-angiogenic chemokines CXCL8 and CCL2, which induce angiogenesis via direct action on endothelial cells. In contrast, CXCL1, CXCL2, CXCL3 and CXCL5 exert pro-angiogenic effects indirectly by recruiting other proangiogenic myeloid cells, including neutrophils [234]. The role of pDCs in angiogenesis has been relatively less characterized than that of cDCs. However, tumor-associated pDCs have been shown to promote angiogenesis in vivo through the production of the proangiogenic cytokines TNF-α and CXCL8 [235]. Finally, DCs may contribute to neovascularization via transdifferentiation. Indeed, DC precursors, recruited to tumor sites via the β-defensin-CCR6 axis, can be transformed into endothelial-like cells with tumor-promoting functions when exposed to VEGF [236].

## Conclusions and perspectives

DCs are the most powerful antigen-presenting cells that activate T cells and the induction of effector antitumor responses. Increasing interest in DC biology and functions has emerged among researchers hoping to exploit their therapeutic potential. However, their high degree of heterogeneity and plasticity and the influence of the surrounding TME can negatively impact their antitumor functions.

Immunotherapy, including both ICI and adoptive transfer therapies, has markedly changed the prospects for cancer patients. However, only a minority of patients exhibit a positive and durable response. Therefore, there is an urgent need to develop combination therapeutic approaches and new treatment strategies to improve the clinical outcomes of cancer patients.

Several strategies are under investigation to restore DC functions and thus improve of antitumor therapy. For instance, targeting CD40 with an agonist antibody has been shown to induce DC activation in colon and pancreatic cancer and to increase antitumor T-cell infiltration [57, 237]. The efficacy of anti-PD-1/PD-L1 treatment positively affected DC functions and induce the antitumor response [238]. Furthermore, blockade of the checkpoint molecule LAG-3 controlled the metabolic program of DCs and their capacity to prime T cells [239]. Another interesting target is the inhibitory receptor TIM3, which has ben shown to control DC antitumor function through ROS-dependent inflammasome activation [240]. TIM3 blockade also enhanced the antitumor response by favoring the colocalization of CD8^+^ T cells and IL-12-producing XCR1^+^ cDC1s [241]. In addition, TIM3 blockade as been shown to regulate cDC activation by promoting cGAS/STING pathway-dependent DNA sensing and the expression of type I IFNs and chemokines [242]. Similarly, blocking TIGIT, an inhibitory receptor expressed by activated CD4^+^ T cells, promoted APC functions and T-cell responses [243, 244]. Finally, although KLRG1 is considered an immune checkpoint marker, and the use of anti-KLRG1 antibodies can prevent tumor metastasis [245], allogenic DC immunization has been shown to exert antitumor effects through the expansion of a KLRG1^+^CD8^+^ T-cell population [246].

Targeting soluble and membrane factors that are known to impair DC functions, such as IL-4, VEGF, AXL and IDO1, have been proven to be effective in mitigating the growth of different tumors, particularly when used in combination with ICI therapy [247–249].

The activation of DCs mediated by tumor cell death induced by chemo- or radiotherapy has recently been exploited against tumors with low immunogenicity [250]. Furthermore, boosting DC functions by using adjuvants such as TLR agonists is an additional therapeutic strategy to mediate DC-mediated antitumor immune responses [251].

A significant challenge remains the development of DC-targeted vaccines for cancer treatment. A promising immunotherapeutic approach involves targeting tumor antigens that are readily cross-presented to increase the efficacy of vaccination. Anti-DEC-205- or anti-CLEC9A-specific antibodies have been shown to increase antigen delivery efficiency and cross-priming functions to induce antitumor immunity [252]. DC cross-presentation is potentiated by type I IFNs, and the use of STING agonists has been shown to enhance the therapeutic response to ICIs [253]. Since the correct localization of DCs is the premise for effective antitumor activity, targeting the selective cDC1 chemokine receptor XCR1 has been shown to be crucial in tumor antigen delivery and T-cell cross-priming. Another option to control tumor growth involves intratumoral inoculation of the ligand XCL1, which increases cDC1 accumulation, and the development of XCL1 engineered variants aimed at improving receptor activity has been recently reported [254]. Notwithstanding their dual role in the modulation of DC antitumor activity, EVs may represent privileged systems for the delivery of antigens to DCs in the context of immunotherapy and DC-based vaccine design, especially TEV-pulsed DCs, which have shown much stronger antitumor activity that than that of DCs loaded with tumor lysates [166]. In addition, novel approaches aimed at maximizing the benefits of increased antigen presentation through TEV engineering systems are under investigation [255].

In conclusion, DCs are the major immune cells involved in presenting tumor antigens and inducing adaptive immune responses. A comprehensive understanding of DC plasticity in response to TME-derived signaling may provide new insights into antitumor therapy. In summary, increasing DC functionality may represent an effective strategy for improving current therapies and developing innovative targeted strategies.
